# Development of the Schedule for Multiple Parallel “Difficult” Peptide Synthesis on Pins

**DOI:** 10.1155/2013/197317

**Published:** 2013-08-20

**Authors:** Ekaterina F. Kolesanova, Maxim A. Sanzhakov, Oleg N. Kharybin

**Affiliations:** Orekhovich Institute of Biomedical Chemistry, Russian Academy of Medical Sciences, 10 Pogodinskaya Ulica, Moscow 119121, Russia

## Abstract

Unified schedule for multiple parallel solid-phase synthesis of so-called “difficult” peptides on polypropylene pins was developed. Increase in the efficiency of 9-fluorenyl(methoxycarbonyl) N-terminal amino-protecting group removal was shown to have a greater influence on the accuracy of the “difficult” peptide synthesis than the use of more efficient amino acid coupling reagents such as aminium salts. Hence the unified schedule for multiple parallel solid-phase synthesis of “difficult” peptides included the procedure for N-terminal amino group deprotection modified by applying a more efficient reagent for the deprotection and the standard procedure of amino acid coupling by carbodiimide method with an additional coupling using aminium salts, if necessary. Amino acid coupling with the help of carbodiimide allows to follow the completeness of the coupling via the bromophenol blue indication, thus providing the accuracy of the synthesis and preventing an overexpenditure of expensive reagents. About 100 biotinylated hepatitis C virus envelope protein fragments, most of which represented “difficult” peptides, were successfully obtained by synthesis on pins with the help of the developed unified schedule.

## 1. Introduction

Development of proteomic and interactome research linked to the mass-spectral detection and amino acid analysis of peptide fragments of proteins requires extensive development of multiple solid-phase peptide synthesis in order to prepare huge sets of peptides used as calibration standards and as affinity ligands for interactome analysis and interaction site mapping [1–5]. These peptide sets are expected to contain up to several hundreds of peptides including those with modified side-chain functional groups, since the analysis of a single tissue sample from a single organism may require the preparation of more than a hundred of the so-called characteristic peptides (unique fragments of proteins under study). The field of peptide scanning usage, which includes multiple parallel peptide syntheses as an obligatory part of the method, also expands. Besides scanning proteins for B- and T-epitope motifs [6–12], kinase phosphorylation and other posttranslational modification sites [13–17], and studies of protease cleavage specificity [16, 18], multiple parallel peptide synthesis is employed for the search of antibacterial peptides [19], receptor peptide ligands [20], and preparation of novel biomaterials based on readily structured peptides and peptoids [21]. Though immunochemical research sometimes allowed the use of peptide preparations with 70–80% purity [22], other previously mentioned fields of peptide employment required highly purified preparations, especially the use as standards for mass spectrometry [1–4]. It necessitates a thorough elaboration of multiple parallel peptide synthesis protocols and development of unified procedures that allow obtaining peptide preparations with maximal contents of target products in the shortest time and with the least material and labor expenses. 

Peptides with so-called “difficult” sequences, prone to the formation of intra- and interchain stable secondary structures, form a group that is characterized by low yields of target products [23, 24]. Hindered amino acid attachment to the growing peptide chain is typical for such peptides resulting in low yields of target products and a lot of byproducts represented by truncated peptides or peptides with gaps. These impurities are often difficult to separate from target products [23–25]. The problems of the “difficult” peptide synthesis are usually solved by (a) adding chaotropic salts or solvents [26, 27], (b) elevation of reaction mixture temperature via conventional heating or microwave irradiation [28–30], (c) use of more efficient catalysts of 9-fluorenyl(methoxycarbonyl) (Fmoc) N-terminal amino-protecting group removal and amino acid acylation [31, 32], and (d) prevention of the aggregation via introducing amido bond modifying groups [25, 33], isoacyl depsipeptide structures [34], and pseudoproline residues [35]. However, addition of chaotropic salts reduces other reagent solubility and hence is undesirable in the case of multiple parallel peptide synthesis on pins, where Fmoc-amino acids are used in high concentrations. Temperature elevation above 60°C is also impossible in this case because of the softening of pins covered with grafted polyethylene. Introduction of amido bond modifications and isoacyl moieties requires the change of coupling conditions only for the peptides, where these modifications are used, hence disrupting the multiple parallel synthesis schedule unification; pseudoproline residues cannot be put everywhere in the peptide sequence. 

Formation of intra- and intermolecular stable secondary structures hampers both the Fmoc removal from *α*-amino-group and Fmoc-amino acid acylation of the growing peptide chain [23, 24]. We studied the influence of the Fmoc removal and Fmoc-amino acid coupling conditions on the accuracy of multiple parallel synthesis of peptides with “difficult” sequences in order to elaborate a unified multiple parallel synthesis schedule that allows the correct synthesis of multiple sets of various peptides on pins. 

## 2. Materials and Methods

Polyethylene pins grafted with *ε*-Fmoc-*α*-Boc-Lys-Pro moiety (DKP pins) and *α*-Fmoc-L-amino acids (except *α*-Fmoc-L-Arg(Pbf)) were from “Mimotopes” (Clayton, Australia). Side-chain functional groups of Fmoc-amino acids were protected with trityl(Trt) (Cys, Ans, and Gln), *t*-butoxy(O*t*Bu) (Asp and Glu), *t-*butyl(*t*Bu) (Ser and Thr), *t-*butyl(oxycarbonyl)(Boc)(Lys and Trp), and 2,2,4,6,7-pentamethyldihydrobenzofuran(Pbf) (Arg). Fmoc-Arg(Pbf), tri(*iso*-propyl)silane (TIS), and 1-[bis(dimethylamino)methylene]-1*H*-1,2,3-triazolo[4,5-b]pyridine-3-oxide hexafluorophosphate (HATU) were from “Sigma-Aldrich” (USA). 1-Hydroxybenzotriazole, 1-[bis (dimethylamino)methylene]-5-chloro-1*H*-benzotriazole-3-oxide hexafluorophosphate (HCTU) was from “Merck Chemicals/Novabiochem” (Nottingham, UK). 1-[Bis(dimethylamino)methylene]-1*H*-benzotriazole-3-oxide hexafluorophosphate (HBTU) was from “Applied Biosystems” (USA). 4-Methyl-piperidine (4MPIP), 1-methyl-piperidinone (NMP), diazabicyclo[5.4.0]undec-7-ene (DBU), trifluoroacetic acid (TFA), 2,4,6-collidine, and D(+)-biotin were from “Acros Organics” (Belgium). N,N′-Di(*iso*-propyl)carbodiimide (DIPC), anisole, and 1,2-ethanedithiol (EDT) were from “Merck” (Darmstadt, Germany). 

Peptides were synthesized on DKP pins by means of multiple parallel solid-phase synthesis in polypropylene 96-well V-bottom plates, well volume 0.32 mL (Matrix, USA). The choice of hepatitis C virus envelope protein sites for scanning and the preparation of the peptide list were described elsewhere [7, 9]. The peptide synthesis from Fmoc-amino acids using DIPC as the condensation catalyst, as described in the “Mimotopes” manual and [22], was chosen as a standard procedure, except that 4-methyl-piperidine (4MPIP) was employed instead of piperidine [36, 37] and NMP was used as the Fmoc removal and amino acid attachment solvent instead of DMF [38]. 

### 2.1. Modifications of Multiple Parallel Peptide Synthesis on Pins

- Fmoc-group removal was performed by 20% 4MPIP and 2% DBU in NMP (here and further v/v% are used). Fmoc amino acid attachment was performed exactly as in the standard procedure. Bromophenol blue was added at 0.05 mg/mL to the reaction mixture for controlling the attachment completeness [39]. Blue color of pins meant that the Fmoc-amino acid attachment reaction should be repeated. The second attachment was carried out using the mixture of 100 mM Fmoc-amino acid, 100 mM HATU, 100 mM HOBT, and 150 mM 2,4,6-collidine in NMP.
- Fmoc-group removal was carried out by 20% 4MPIP (the standard procedure). Fmoc-amino acid attachment was performed using the mixture of 100 mM Fmoc-amino acid, 100 mM HATU, 100 mM HOBT, and 150 mM 2,4,6-collidine in NMP.
- Fmoc-group removal was carried out as in the standard procedure. Fmoc-amino acids were attached as in (B), except that HBTU was used instead of HATU in the same concentration.
- Fmoc-group removal was carried out as in the standard procedure. Fmoc-amino acids were attached as in (B), except that HCTU was used instead of HATU in the same concentration. The repeated Fmoc-amino acid attachment was not carried out in modifications (B)–(D). The peptides that were used for testing the previously mentioned synthesis modifications are listed in Table 1.

Peptide biotinylation was performed on pins as described earlier [7]. Removal of side-chain protecting groups was carried out by the mixture trifluoroacetic acid- (TFA-) 1,2-ethanedithiol- (EDT-) water-tri(*iso*-propyl)silane- (TIS-) anisole (915 : 25 : 25 : 10 : 25, v/v) for 4 hours at room temperature. Peptides were detached from pins into 40% acetonitrile in 0.1 M ammonium bicarbonate, pH 8.4 (0.8 mL per pin) by fourfold 15 min ultrasonication at 40°C. 

Matrix-assisted laser desorption-ionization time-of-flight mass spectrometric analysis (MALDI-TOF MS) of peptides was carried out on MicroFlex (“Bruker Daltonics,” Germany) equipped with nitrogen laser (*λ* = 337 nm), in a reflectron mode with 25 kV acceleration potential. *α*-Cyano-4-hydroxycinnamic acid was used as a matrix. Samples were applied in triplicate onto MSP AnchorChip 600/96 plate via 1 mL drop layering on the matrix. 

HPLC analysis of peptide preparations upon the modification of multiple parallel synthesis procedure development was performed on Agilent 1200 Series HPLC system (Agilent Technologies, USA) equipped with Zorbax 300SB-C18 (3.5 *μ*m) 1.0 mm × 150 mm column (Agilent Technologies), elution with 2–80% gradient of 0.1% HCOOH in acetonitrile in 0.1% HCOOH water solution starting 5 min following injection, elution rate 50 *μ*L/min. Target peptide and byproduct detection and analysis were performed by ESI-MS and tandem mass-spectrometry (MS/MS) by collision-induced dissociation (CID) via Ar atom (25 eV) bombardment on Apex Qe Fourier transform ion cyclotron resonance mass spectrometer (“Bruker Daltonics,” Germany). Mass spectra were analyzed with the help of FlexControl software (“Bruker Daltonics”). 

The list of peptides for synthesis and step-by-step schedule of multiple parallel synthesis were prepared with the help of PEPMAKER software (“Mimotopes”). Possible problems in each peptide synthesis were analyzed with the help of PINSOFT2 software (“Mimotopes”).

## 3. Results and Discussion

Tables 1 and 2 comprise the lists of biotinylated peptides containing octapeptide fragments of HCV envelope proteins that were synthesized in this work. PINSOFT2 analysis of the peptide primary structures showed that some of the listed peptides have amino acid sequences, which are prone to the aggregation during the solid-phase synthesis: amino acid residues with *β*-methyl groups located in a row, amino acid residues with hydrophobic side chains or bulky side-chain protecting groups located one through one, and a big proportion of Gly residues, that are capable to form inter- and intramolecular hydrogen bonds [23, 24]. 

**Figure 1:**
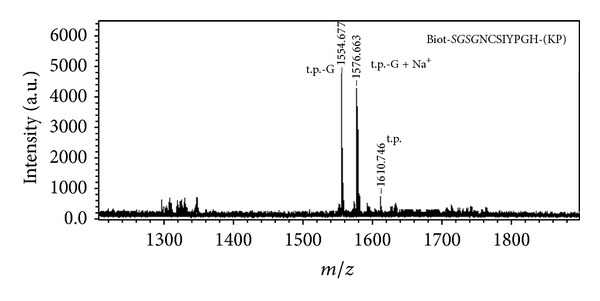
MALDI-TOF MS of the preparation of peptide Biotinyl-*SGSG*NCSIYPGH-(KP) obtained with the help of the standard multiple parallel peptide synthesis schedule on pins. t.p.: target peptide; t.p.-G: Biotinyl-*SGS*NCSIYPGH-(KP).

Preliminary synthesis experiments with further MALDI-TOF MS and HPLC with MS/MS analysis (ESI followed by CIS) showed that all preparations of peptides with Gly-Gly fragment and some with Gly-X (where X = amino acid residue with Trt side-chain protecting group, mainly N and Q) just following the (Lys-Pro) diketopiperazine unit prepared by the standard procedure of multiple parallel peptide synthesis on pins (see Section 2 and [22]) contained a large portion of byproducts lacking Gly residue in this pair. Some preparations contained byproducts lacking residues inside –SGSG– linker group, and many preparations contained short truncated and also lacking certain amino acid residues peptides with nonremoved Fmoc group (mass peaks from 1280 to 1340 Da in Figure 1). Preparations of peptides nos. 86–88, 90–91 contained only trace amounts of target substances. 

Hence a modification of the standard synthesis schedule was needed to achieve the correct synthesis of peptides listed in Tables 1 and 2. In particular, it was necessary to reveal whether the activation of Fmoc removal from growing peptide chain or Fmoc amino acid acylation could influence the purity of the target peptide, especially the absence of byproducts lacking 1-2 amino acid residues compared to the target peptide, more efficiently. Tertiary amine DBU was used as a more efficient catalyst of the hydroxycarbonyl dibenzofulvene detachment from the peptide *α*-amino group, in addition to 4MPIP [31]. Since DBU actively catalyzes the formation of aspartimides, Asp piperidines, Asp epimerization, and Asn dehydration [23, 31, 36], it was not added to the Fmoc removal reagent after Asp or Asn introduction to the growing peptide chains. HATU, HBTU, and HCTU were employed as more efficient acylation activators, among which HATU is the most and HBTU is the least (close to DIC) efficient.

**Figure 2:**
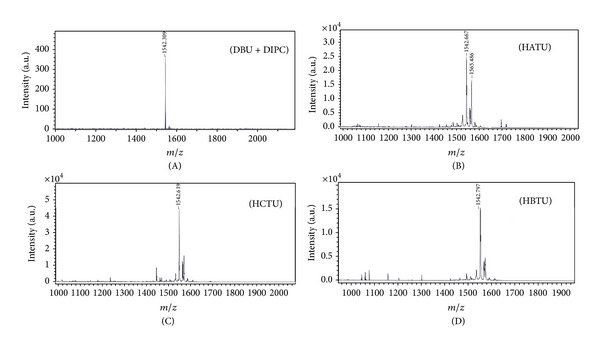
MALDI-TOF MS of preparations of peptide Biotinyl-SGSGNTKLMGGT-(KP) (mol. mass 1542.3) obtained with the help of modifications (a), (b), (c), and (d) of the standard schedule of parallel peptide synthesis on pins (see Section 2 for details).

Table 1 shows HPLC and ESI MS and MS/MS analysis results of the final preparations of 5 peptides synthesized using modifications (A)–(D) of the standard multiple parallel peptide synthesis procedure on pins. Synthesis of these peptides by the standard procedure gave incorrect results (see Table 1). One can see that the application of the more efficient catalyst of Fmoc removal resulted in peptide preparation containing less byproducts lacking one or several amino acid residues compared to the target products than the application of aminium salts as acylation activators, in general. The latter also improved the results of the synthesis of peptides with “difficult” sequences; however, the use of aminium salts as acylation activators requires the addition of a tertiary amine (in our case, 2,4,6-collidine) to the reaction mixture, hence excluding the possibility to control the completeness of Fmoc-amino acid attachment to the growing peptide chain with bromophenol blue. Moreover, MS ESI and MS/MS analyses revealed noticeable (though not exceeding 5% of the target product mass peak intensity) mass peaks of Fmoc-containing truncated byproduct peptides in preparations obtained with modifications (B)–(D), but not (A) (see Figure 2). Hence the modification (A) of the standard multiple parallel solid-phase peptide synthesis schedule was used for the preparation of hepatitis C virus (HCV) envelope protein fragments for further B-epitope mapping and characterization of the prepared anti-HCV envelope antibodies.  Table 2 contains the structures of the synthesized peptides and results of MALDI-TOF MS analyses of their unpurified preparations. One can see that the enhancement of the efficiency of Fmoc removal resulted in much more correct synthesis of “difficult” peptides that we could not obtain in good purity and even could not obtain at all (peptides 86–88, 90, 91) with the help of the standard schedule.

## 4. Conclusion

In general, enhancement of the Fmoc removal efficiency in the parallel solid-phase peptide synthesis on pins showed a greater potential in the improvement of “difficult” peptide synthesis than the enhancement of the acylation stage efficiency. Moreover, the use of the standard DIC activation procedure better helped to control the Fmoc amino acid coupling completeness by bromophenol blue indication [39], thus excluding the overexpenditure of Fmoc amino acids, acylation reagents, and time, compared to the procedure with all coupling stages repeated in order to achieve the correct synthesis of target peptides. One should take into account that not only the aggregation of peptide chains due to interchain hydrogen bonds makes the peptide sequence difficult for synthesis. In our case the most difficult stage was the attachment of the fourth (or, maybe, fifth) residue from the C-terminus, when no peptide aggregation was supposed to occur [23]. However, the presence of rather long flexible structures (side chain of Lys, to which the first amino acid residue of HCV protein fragment was attached and Gly in the second position from the peptide C-terminus) and Pro known to induce the turn formation could result in such turn of the growing peptide chain, augmented by the possible formation of intrachain H-bonds and hydrophobic interactions that hid the peptide N-terminal thus embarrassing its both Fmoc deblocking and further acylation. In this case, the sole enhancement of the amino acid coupling efficiency was shown to be less productive than the use of more efficient Fmoc deblocking reagent [40, 41]. Also, one could suppose that deblocking of the peptide N-terminus would disrupt the structure that hampered the further growing chain acylation, at least for short peptide chains. The same effect is frequently observed in the synthesis of peptides with Pro-Pro-, Val-Val-, -(Val-Thr(Ile))-pairs, Pro close to Val or Thr, and so forth, which are prone to *β*-turn formation [42]. Hence the DBU addition to 4MPIP or piperidine can be recommended as a modification of the standard procedure of multiple parallel peptide synthesis on pins, in order to obtain a unified procedure that allows the correct synthesis of “difficult” peptides. The only problem of DBU usage is its ability to catalyze aspartimide formation from Asp [36]. However, certain side-chain carboxyl protection groups greatly reduce [43–46], and Asp-X peptide bond modifications exclude this side reaction [25, 33, 46], hence permitting the use of more efficient Fmoc removal catalyst in a greater set of difficult synthesis cases. 

## Figures and Tables

**Table 1:** Peptides synthesized with the help of modifications (A)–(D) (see Section 2) of the standard multiple parallel solid-phase peptide synthesis schedule and MALDI-TOF MS peak lists of their nonpurified preparations.

Peptide number	Amino acid sequence and calculated molecular mass (Da) of the peptide^1^	Molecular masses of peptide products, obtained by modifications (A)–(D) of the standard multiple parallel synthesis schedule, and relative intensities of the corresponding ion peaks (HPLC with ESI-MS detection)^2^			
		(A)	(B)	(C)	(D)
1a	Biotin*-SGSG * **T**TKVIGGT-(KP) 1497,8	1497.6	1497.5	1497.4	1497.7 1396.5 (−T; 17%)

2a	Biotin-*SGSG*QTR**T**T**G**GS-(KP) 1528,7	1528.5	1528.6	1528.4	1528.6 1427.5 (−T; 20%) 1471.4 (−G; 13%) 1370.2 (−G − T; 7%)

3a	Biotin-*SGSG*N**T**KLMGGT-(KP) 1542,8	1542.3	1542.7 1558.8 (Met(O))	1542.8 1558.7 (Met(O)) 1441.4 (−T; 15%)	1542.6 1558.6 (Met(O)) 1485.4 (−G; 10%) 1080.7 (Fmoc-TKLMGT-(KP); 5%)

4a	Biotin-*SGSG*NNYVT**G**GA-(KP) 1516,7	1516.3	1516.4	1516.4	1516.5 1459.7 (−G; 15%)

5a	Biotin-***S*** *GSG*D**T**RVV**G**GQ-(KP) 1552,8	1552.6	1552.6 1495.5 (−G; 10%)	1552.6 1495.5 (−G; 17%) 1465.5 (−S; 10%)	1552.7 1451.5 (−T; 17%) 1495.5 (−G; 15%)

^1^Linker sequence between the octapeptide fragment of HCV envelope protein and biotin moiety is marked by italics. *ε*-(Lys-Pro)-Diketopiperazine moiety is shown in brackets.

^2^The intensity of the target product mass peak is taken as 100% in each case. Mass peaks with intensities not less than 5% of target product mass peak intensities are only listed. Lacking residues are shown as (−*X*) and in bold in peptide sequences.

**Table 2:** Peptides synthesized by the modification (1) of the standard parallel solid-phase peptide synthesis schedule on pins.

Peptide number	Peptide sequence	HCV protein source of octapeptide fragment	Calculated molecular mass of peptide, *D*_*a*_	Masses of molecular ions in MALDI-TOF mass spectra, *D*_*a*_
1	Biotin-*SG * ***S*** *G*PGCVPCVR-(KP)	E1	1551.9	1551.8
2	Biotin-*SGSG*GCVPCVRE-(KP)	E1	1583.9	1583.8
3	Biotin-*SGSG*YVGDLCGS-(KP)	E1	1534.8	1556.3 (+Na^+^)
4	Biotin-*SGSG*VGDLCGSV-(KP)	E1	1470.7	1492.7 (+Na^+^)
5	Biotin-*SGSG*GDLCGSVF-(KP)	E1	1518.8	1540.7 (+Na^+^)
6	Biotin-*SGSG*DLCGSVFL-(KP)	E1	1574.9	1596.8 (+Na^+^)
7	Biotin-*SGSG*QLFTFSPR-(KP)	E1	1717.0	1716.9
8	Biotin-*SGSG*QDCNCSIY-(KP)	E1	1666.9	1688.7 (+Na^+^)
9	Biotin-*SGSG*CNCSIYPG-(KP)	E1	1577.8	1599.6 (+Na^+^)
10	Biotin-*SG * ***SG***NCSIYPGH-(KP)	E1	1611.9	1611.3 1633.3 (+Na^+^)
11	Biotin-*SGSG*AWDMMMNW-(KP)	E1	1806.2	1827.7 (+Na^+^)
12	Biotin-*SGSG*WDMMMNWS-(KP)	E1	1822.2	1843.7 (+Na^+^)
13	Biotin-*SGSG*DMMMNWSP-(KP)	E1	1733.1	1770.7 (+Na^+^; +O) 1754.7 (+Na^+^) 1786.6 (+Na^+^; +2O)
14	Biotin-*SGSG*MMMNWSPT-KPP	E1	1719.1	1756.7 (+Na^+^; +O) 1772.7 (+Na^+^; +2O) 1740.7 (+Na^+^) 1788.7 (+Na^+^; +3O)
15	Biotin-*SGSG*AGAHWGVL-(KP)	E1	1531.8	1553.8 (+Na^+^) 1531.8
16	Biotin-*SGSG*GAHWGVLA-(KP)	E1	1531.8	1553.8 (+Na^+^) 1531.8
17	Biotin-*SGSG*AHWGVLAG-(KP)	E1	1531.8	1553.8 (+Na^+^) 1531.8
18	Biotin-*SGSG*SMVGNWAK-(KP)	E1	1613.9	1635.8 (+Na^+^) 1613.8
19	Biotin-*SGSG*MVGNWAKV-(KP)	E1	1625.9	1625.8 1647.8 (+Na^+^) 1641.8 (+O) 1663.8 (+Na^+^; +O)
20	Biotin-*SGSG*VGNWAKVL-(KP)	E1	1607.9	1629.9 (+Na^+^) 1607.9
21	Biotin-*SGSG*INTNGSWH-(KP)	E2	1649.8	1671.7 (+Na^+^) 1649.7
22	Biotin-*SGSG*N**T**NGSWHI-(KP)	E2	1649.8	1649.8 1671.8 (+Na^+^)
23	Biotin-*SGSG*TNGSWHIN-(KP)	E2	1649.8	1649.8 1671.8 (+Na^+^)
24	Biotin-*SGSG*NGSWHINR-(KP)	E2	1704.9	1704.8
25	Biotin-*SGSG*ALNCNDSL-(KP)	E2	1570.8	1592.7 (+Na^+^)
26	Biotin-*SGSG*PVVVGTTD-(KP)	E2	1508.8	1530.8 (+Na^+^)
27	Biotin-*SGSG*VVVGTTDR-(KP)	E2	1567.8	1567.8
28	Biotin-*SGSG*WGENETDV-(KP)	E2	1670.8	1692.8 (+Na^+^)
29	Biotin-*SGSG*GNWFGCTW-(KP)	E2	1691.9	1709.8 (+H_2_O)
30	Biotin-*SGSG*NWFGCTWM-(KP)	E2	1766.1	1787.8 (+Na^+^)
31	Biotin-*SGSG*FGCTWMNS-(KP)	E2	1666.9	1688.7 (+Na^+^)
32	Biotin-*SGSG*KCGSGPWL-(KP)	E2	1535.8	1557.7 (+Na^+^) 1535.7
33	Biotin-*SGSG*TGFTKTCG-(KP)	E2	1568.9	1568.8
34	Biotin-*SGSG*CGSGPWLT-(KP)	E2	1541.8	1563.7 (+Na^+^)
35	Biotin-*SGSG*GSGPWLTP-(KP)	E2	1535.8	1557.7 (+Na^+^)
36	Biotin-*SGSG*SGPWLTPR-(KP)	E2	1634.9	1634.8
37	Biotin-*SGSG*GPWLTPRC-(KP)	E2	1651.0	1650.9
38	Biotin-*SGSG*HYPCTVNF-(KP)	E2	1702.0	1701.8 1723.8 (+Na^+^)
39	Biotin-*SGSG*RMYVGGVE-(KP)	E2	1631.9	1631.9 1647.8 (+O)
40	Biotin-*SGSG*TGFTKTCG-(KP)	E2	1612.9	1612.8 1634.8 (+Na^+^)
41	Biotin-*SGSG*YVGGVEHR-(KP)	E2	1637.9	1637.9 1659.8 (+Na^+^)
42	Biotin-*SGSG*VGGVEHRL-(KP)	E2	1587.8	1587.8
43	Biotin-*SGSG*AACNWTRG-(KP)	E2	1599.8	1599.8
44	Biotin-*SGSG*ACNWTRGE-(KP)	E2	1657.9	1657.7
45	Biotin-*SGSG*CNWTRGER-(KP)	E2	1743.0	1742.8
46	Biotin-*SGSG*NWTRGERC-(KP)	E2	1743.0	1742.8
47	Biotin-*SGSG*LEDRDRSE-(KP)	E2	1740.0	1740.8 1762.8 (+Na^+^)
48	Biotin-*SGSG*EDRDRSEL-(KP)	E2	1740.0	1740.8 1762.8 (+Na^+^)
49	Biotin-*SGSG*DRDRSELS-(KP)	E2	1698.9	1698.7
50	Biotin-*SGSG*RDRSELSP-(KP)	E2	1680.9	1680.8
51	Biotin-*SGSG*DRSELSPL-(KP)	E2	1637.9	1637.8 1659.8 (+Na^+^)
52	Biotin-*SGSG*RSELSPLL-(KP)	E2	1636.0	1635.8
53	Biotin-*SGSG*IHLHQNIV-(KP)	E2	1570.9	1592.7 (+Na^+^)
54	Biotin-*SGSG*TTLPALST-(KP)	E2	1524.8	1546.7 (+Na^+^)
55	Biotin-*SGSG*TLPALSTG-(KP)	E2	1480.7	1502.7 (+Na^+^)
56	Biotin-*SGSG*IHLHQNIV-(KP)	E2	1695.0	1694.8 1716.8 (+Na^+^)
57	Biotin-*SGSG*SDLPALST-(KP)	E2	1524.8	1546.7 (+Na^+^)
58	Biotin-*SGSG*TPMPALST-(KP)	E2	1538.9	1576.6 (+Na^+^; +O)
59	Biotin-*SGSG*DLPALSTG-(KP)	E2	1494.7	1516.6 (+Na^+^)
60	Biotin-*SGSG*PMPALSTG-(KP)	E2	1494.8	1532.6 (+Na^+^; +O)
61	Biotin-*SG * ***S*** *G*ETLSVGGS-(KP)	E2	1470.7	1492.4 (+Na^+^)
62	Biotin-*SG * ***S*** *G*ETIVTGGT-(KP)	E2	1498.7	1520.6
63	Biotin-*SG * ***S*** *G*ETAVSGGT-(KP)	E2	1442.6	1464.4 (+Na^+^)
64	Biotin-*SG * ***S*** *G*ET**R**VSGGT-(KP)	E2	1527.7	1527.3
65	Biotin-*SGSG*GTYTTGGA-(KP)	E2	1448.6	1470.6 (+Na^+^)
66	Biotin-*SGSG*TTYTTGGS-(KP)	E2	1508.7	1530.4 (+Na^+^)
67 (1a)	Biotin-*SGSG*TTKVIGGT-(KP)	E2	1497.8	1497.6
68	Biotin-*SGSG*GT**R**TMGGA-(KP)	E2	1471.7	1471.5
69	Biotin-***S*** *G * ***SG***GTHVTGGS-(KP)	E2	1436.6	1436.4
70	Biotin-*SGSG*GT**R**VSGGT-(KP)	E2	1455.7	1455.4
71	Biotin-***SGS*** *G*STHVTGGA-(KP)	E2	1450.6	1450.5
72	Biotin-*SG * ***S*** *G*STYTTGGS-(KP)	E2	1494.7	1516.3 (+Na^+^)
73	Biotin-*SG * ***S*** *G*STTITGGS-(KP)	E2	1444.6	1466.5 (+Na^+^)
74	Biotin-*SGSG*ST**R**VTGGA-(KP)	E2	1469.7	1469.5
75	Biotin-***SGS*** *G*QTHTTGGS-(KP)	E2	1528.7	1528.8 1551.1 (+Na^+^)
76	Biotin-*SGSG*GTRVSGGT-(KP)	E2	1509.7	1509.4 1531.3 (+Na^+^)
77	Biotin-*SGSG*QTYVTGGA-(KP)	E2	1517.7	1539.4 (+Na^+^)
78	Biotin-*SGSG*KTYTTGGA-(KP)	E2	1519.7	1519.7 1541.7 (+Na^+^)
79	Biotin-*SG * ***S*** *G*KTHVTGGS-(KP)	E2	1507.7	1507.5
80	Biotin-*SG * ***S*** *G * **R**THVTGGS-(KP)	E2	1535.7	1535.7
81	Biotin-*SGSG*GTYVTGGA-(KP)	E2	1446.6	1535.7
82	Biotin-*SGS * ***G*R**T**R**LTGGN-(KP)	E2	1595.8	1595.6
83	Biotin-*S* *G* *SG * **R**TKTIGGT-(KP)	E2	1554.8	1554.6
84	Biotin-*SGSG*ATYTTGGA-(KP)	E2	1462.7	1462.4 1484.5 (+Na^+^)
85	Biotin-*SGSG*ATHVTGGT-(KP)	E2	1464.7	1464.3
86	Biotin-*SG * ***S*** *G*NTYTTGGS-(KP)	E2	1521.7	1543.4 (+Na^+^)
87 (3a)	Biotin-*SG * ***S*** *G*NTKLMGGT-(KP)	E2	1542.8	1542.5
88	Biotin-*SG * ***S*** *G*NT**R**TTGGT-(KP)	E2	1528.7	1528.6
89 (4a)	Biotin-*SGSG*NNYVTGGA-(KP)	E2	1516.7	1538.3 (+Na^+^)
90	Biotin-*SGSG*DTHVTGGS-(KP)	E2	1494.6	1494.4
91	Biotin-*SG * ***S*** *G*HT**R**TTGGA-(KP)	E2	1521.7	1521.4
92 (5a)	Biotin-*SGSG*DT**R**VVGGQ-(KP)	E2	1552.8	1552.6
93	Biotin-*SG * ***SG***HTYTTGGT-(KP)	E2	1558.7	1558.7
94	Biotin-*SG * ***SG***HTHTTGGV-(KP)	E2	1530.7	1530.4
95	Biotin-*SG * ***SG***HTHVTGGV-(KP)	E2	1528.7	1528.5
96	Biotin-*SG * ***S*** *G*DTYTTGGS-(KP)	E2	1522.7	1544.4 (+Na^+^)

Amino acid residues, for which repeated coupling was performed while using modification (A) of the standard procedure, are shown in bold.
